# *MYC* Deregulation in Gastric Cancer and Its Clinicopathological Implications

**DOI:** 10.1371/journal.pone.0064420

**Published:** 2013-05-22

**Authors:** Carolina Rosal Teixeira de Souza, Mariana Ferreira Leal, Danielle Queiroz Calcagno, Eliana Kelly Costa Sozinho, Bárbara do Nascimento Borges, Raquel Carvalho Montenegro, Ândrea Kely Campos Ribeiro dos Santos, Sidney Emanuel Batista dos Santos, Helem Ferreira Ribeiro, Paulo Pimentel Assumpção, Marília de Arruda Cardoso Smith, Rommel Rodríguez Burbano

**Affiliations:** 1 Laboratório de Citogenética Humana, Instituto de Ciências Biológicas, Universidade Federal do Pará, Belém, Pará, Brazil; 2 Departamento de Ortopedia e Traumatologia, Universidade Federal de São Paulo, São Paulo, São Paulo, Brazil; 3 Disciplina de Genética, Departamento de Morfologia e Genética, Universidade Federal de São Paulo, São Paulo, São Paulo, Brazil; 4 Laboratório de Genética Humana e Medica, Instituto de Ciências Biológicas, Universidade Federal do Pará, Belém, Pará, Brazil; 5 Núcleo de Pesquisa em Oncologia, Universidade Federal do Pará, Belém, Pará, Brazil; National Cancer Center, Japan

## Abstract

Our study investigated the relationship between *MYC* alterations and clinicopathological features in gastric cancers. We evaluated the effect of *MYC* mRNA expression and its protein immunoreactivity, as well as copy number variation, promoter DNA methylation, and point mutations, in 125 gastric adenocarcinoma and 67 paried non-neoplastic tissues. We observed that 77% of the tumors presented MYC immunoreactivity which was significantly associated with increased mRNA expression (*p*<0.05). These observations were associated with deeper tumor extension and the presence of metastasis (*p*<0.05). MYC protein expression was also more frequently observed in intestinal-type than in diffuse-type tumors (*p*<0.001). Additionally, *MYC* mRNA and protein expression were significantly associated with its copy number (*p*<0.05). The gain of *MYC* copies was associated with late-onset, intestinal-type, advanced tumor stage, and the presence of distant metastasis (*p*<0.05). A hypomethylated *MYC* promoter was detected in 86.4% of tumor samples. *MYC* hypomethylation was associated with diffuse-type, advanced tumor stage, deeper tumor extension, and the presence of lymph node metastasis (*p*<0.05). Moreover, eighteen tumor samples presented at least one known mutation. The presence of *MYC* mutations was associated with diffuse-type tumor (*p*<0.001). Our results showed that *MYC* deregulation was mainly associated with poor prognostic features and also reinforced the presence of different pathways involved in intestinal-type and diffuse-type gastric carcinogenesis. Thus, our findings suggest that *MYC* may be a useful marker for clinical stratification and prognosis.

## Introduction

Gastric cancer is the fourth most frequent type of cancer and remains the second leading cause of cancer-related death worldwide [1]. This cancer is usually diagnosed at advanced stages and the single curative therapy available requires surgical resection [2]. Thus, gastric cancer is a serious public health problem in the world. An improved understanding of the biology of this neoplasm is critical and may be useful to guide patient management, as well as to develop new therapeutic options.

*MYC* is one of the most studied oncogenes stemming from its association with a large number of diseases [3]. MYC plays a role in several fundamental functions of cell biology, including the regulation of cell growth and proliferation, metabolism, differentiation, apoptosis, and angiogenesis (for review see [4], [5]). Hence, MYC is an integrator of extracellular and intracellular signals, and its cellular phenotype is dependent on tissue location [6], [7]. Not surprisingly, deregulation of MYC functions contributes to the tumor phenotype.

*MYC* deregulation due to gene amplification [8], [9], chromosomal translocation or insertion [10], [11], mutations [12], and epigenetic modifications [13], [14], has been reported in different types of cancers, especially in gastric cancer. MYC expression is often elevated or deregulated in human neoplasms [4], and seems to be at the crossroad of several important pathways and processes involved in carcinogenesis [15], being a key event in gastric carcinogenesis [9]. Previously, our group demonstrated that *MYC* mRNA expression and copy number increases during the sequential steps of intestinal-type gastric carcinogenesis in a non-human primate model [16], suggesting that *MYC* may be involved in gastric tumor initiation and progression.

The understanding of *MYC* biology is of paramount importance to elucidate its role in the pathogenesis of gastric cancer. Up to date, there is no study correlating *MYC* mutation, amplification, protein/mRNA levels, and methylation in this neoplasia. Here, we evaluated the relationship between *MYC* alterations and clinicopathological features in gastric cancer. In addition, *MYC* mRNA expression and protein immunoreactivity, as well as several molecular mechanisms previously related to its deregulation as copy number variation (CNV), mutation, and DNA methylation, were analyzed in the same set of gastric cancer samples.

## Materials and Methods

### Ethics Statement

All samples were derived with written informed consent and approval from the University Hospital (Belém, Pará, Brazil) ethical review boards (protocol number: 142004).

### Clinical Samples

125 gastric adenocarcinoma and 67 corresponding non-neoplastic gastric tissues (control samples) were obtained surgically from patients of the João de Barros Barreto University Hospital in Pará State, Brazil. All subjects were not exposed to either chemotherapy or radiotherapy before surgery. Gastric tumors were classified according to Lauren [17] and tumors were staged using standard criteria by TNM staging [18]. The clinicopathological features are shown in table 1 and 2.

**Table 1:** Clinicopathological characteristics, MYC immunoreactivity, DNA methylation and point mutations in gastric cancer samples.

Variable (N)	Protein immunoreactivity					DNA methylation					Point mutations				
	Positive [N(%)]*	Negative [N(%)]	*p*-value	OR	CI 95%	Hypomethylated [N(%)]*	Partial methylated [N(%)]	*p*-value	OR	CI 95%	Present [N(%)]*	Absent [N(%)]	*p*-value	OR	CI 95%
**Gender**															
Female (40)	29 (23.2%)	11 (8.8%)	0.641	1.241	0.501–3.079	33 (26.4%)	7 (5.6%)	0.480	1.468	0.506–4.260	6 (4.8%)	34 (27.2%)	0.247	0.501	0.156–1.614
Male (85)*	67 (53.6%)	18 (14.4%)				75 (60%)	10 (8%)				7 (5.6%)	78 (62.4%)			
**Onset (years)**															
≤45 (18)	10 (8%)	8 (6.4%)	0.026**	3.276	1.152–9.315	14 (11.2%)	4 (3.2%)	0.257	2.066	0.590–7.236	2 (1.6%)	16 (12.8%)	0.915	0.917	0.186–4.526
>45 (107)*	86 (68.8%)	21 (16.8%)				94 (75.2%)	13 (10.4%)				11 (8.8%)	96 (76.8%)			
**Tumor location**															
Non-cardia (73)	58 (46.4%)	15 (12%)	0.262	0.604	0.250–1.457	63 (50.4%)	10 (8%)	0.929	0.953	0.333–2.732	7 (5.6%)	66 (52.8%)	0.732	1.225	0.385–3.899
Cardia (52)*	38 (30.4%)	14 (11.2%)				45 (36%)	7 (5.6%)				6 (4.8%)	46 (36.8%)			
**Histologic subtype**															
Diffuse-type (54)	31 (24.8%)	23 (18.4%)	<0.001**	7.856	2.803–22.013	52 (41.6%)	2 (1.6%)	0.007**	0.117	0.025–0.556	12 (9.6%)	42 (33.6%)	0.004**	0.046	0.006–0.373
Intestinal-type (71)*	65 (52%)	6 (4.8%)				56 (44.8%)	15 (12%)				1 (0.8%)	70 (56%)			
**Stage**															
Early (8)	4 (3.2%)	4 (3.2%)	0.593	1.546	0.312–7.652	4 (3.2%)	4 (3.2%)	0.033**	6.602	1.162–37.501	2 (1.6%)	6 (4.8%)	0.135	0.204	0.025–1.643
Advanced (117)*	92 (73.6%)	25 (20%)				104 (83.2%)	13 (10.4%)				11 (8.8%)	106 (84.8%)			
**Tumor invasion**															
T1/T2 (30)	16 (12.8%)	14 (11.2%)	0.045**	2.975	1.027–8.623	21 (16.8%)	9 (7.2%)	0.022**	4.752	1.257–17.965	3 (2.4%)	27 (21.6%)	0.978	1.023	0.205–5.101
T3/T4 (95)*	80 (64%)	15 (12%)				87 (69.6%)	8 (6.4%)				10 (8%)	85 (68%)			
**Lymph node metastasis**															
Absent (12)	0 (0%)	12 (9.6%)	0.998	<0.001	0.000	7 (5.6%)	5 (4%)	0.032**	5.120	1.149–22.814	0 (0%)	12 (9.6%)	0.999	<0.001	0.000
Present (113)*	96 (76.8%)	17 (13.6%)				101 (80.8%)	12 (9.6%)				13 (10.4%)	100 (80%)			
**Distant metastasis**															
Absent (66)	39 (31.2%)	27 (21.6%)	<0.001**	17.682	3.914–79.882	55 (44%)	11 (8.8%)	0.439	1.537	0.517–4.571	3 (2.4%)	63 (50.4%)	0.032**	4.492	1.141–17.679
Present (59)*	57 (45.6%)	2 (1.6%)				53 (42.4%)	6 (4.8%)				10 (8%)	49 (39.2%)			
**Protein immunoreactivity**															
Negative (29)	-	-	-	-	-	22 (17.6%)	7 (5.6%)	0.151	2.283	0.739–7.056	2 (1.6%)	27 (21.6%)	0.486	0.562	0.112–2.835
Positive (96)*	-	-				86 (68.8%)	10 (8%)				11 (8.8%)	85 (68%)			
**DNA methylation**															
Partial methylated (17)	10 (8%)	7 (5.6%)	0.142	2.303	0.755–7.025	-	-	-	-	-	0 (0%)	17 (13.6%)	0.998	<0.001	0.000
Hypomethylated (108)*	86 (68.8%)	22 (17.6%)				-	-				13 (10.4%)	95 (76%)			

* Reference group for logistic regression analysis;

** Differentially expressed between groups, p<0.05.

N: number of samples; OR: odds ratio; CI: confidence interval.

**Table 2:** Clinicopathological characteristics, *MYC* mRNA expression, copy number and percentage of amplification in gastric cancer samples.

Variable (N)	RQ				Copy number				% of amplification (FISH)			
	Mean±SD	*p*-value	η^2^	OP	Mean±SD	*p*-value	η^2^	OP	Mean±SD	*p*-value	η^2^	OP
**Gender**												
Female (16)	3.42±1.03	0.410	0.015	0.129	4.50±1.55	0.402	0.015	0.132	70.69±8.03	0.658	0.004	0.072
Male (33)	3.37±0.92				4.48±1.30				72.77±7.81			
**Onset (years)**												
≤45 (5)	2.63±0.82	0.060	0.073	0.472	3.2±0.45	0.025*	0.103	0.622	60.4±5.21	<0.001*	0.257	0.976
>45 (44)	3.47±0.93				4.63±1.37				73.42±6.98			
**Tumor location**												
Non-cardia (28)	3.57±0.93	0.108	0.055	0.362	4.75±1.53	0.102	0.057	0.372	72.52±8.36	0.585	0.007	0.084
Cardia (21)	3.14±0.93				4.14±1.06				71.52±7.31			
**Histologic subtype**												
Diffuse-type (21)	3.35±0.67	0.723	0.003	0.064	3.81±0.75	0.009*	0.139	0.762	68.55±8.77	0.037*	0.091	0.557
Intestinal-type (28)	3.41±1.12				5.00±1.52				74.75±5.98			
**Stage**												
Early (2)	2.09±0.14	0.202	0.035	0.245	3.00±0.00	0.546	0.008	0.091	54.5±6.36	0.037*	0.091	0.558
Advanced (47)	3.44±0.93				4.55±1.37				72.84±7.04			
**Tumor invasion**												
T1/T2 (11)	2.55±0.84	0.006*	0.152	0.801	3.55±0.69	0.200	0.035	0.246	60.82±6.36	<0.001*	0.401	1.000
T3/T4 (38)	3.63±0.84				4.76±1.40				75.36±4.53			
**Lymph node metastasis**												
Absent (5)	2.27±0.32	0.023*	0.107	0.632	3.20±0.45	0.143	0.046	0.308	36.60±6.94	0.179	0.039	0.267
Present (44)	3.51±0.91				4.64±1.37				73.06±7.43			
**Distant metastasis**												
Absent (23)	2.50±0.48	<0.001*	0.788	1.000	3.61±0.66	<0.001*	0.356	0.999	68.20±7.29	0.001*	0.221	0.942
Present (26)	4.16±0.41				5.27±1.37				75.54±6.75			
**Protein immunoreactivity**												
Negative (11)	2.56±0.44	0.003*	0.178	0.870	3.27±0.47	0.003*	0.177	0.869	65.45±8.37	0.009*	0.139	0.759
Positive (38)	3.63±0.92				4.84±1.35				74.01±6.66			
**DNA methylation**												
Hypomethylated (42)	3.40±0.91	0.802	0.001	0.057	4.45±1.37	0.283	0.025	0.186	72.40±7.40	0.806	0.001	0.057
Partial methylated (7)	3.33±1.24				4.71±1.5				70.21±10.73			
**Point mutations**												
Present (7)	3.73±0.79	0.107	0.055	0.363	4.43±0.79	0.614	0.006	0.079	72.00±10.83	0.216	0.033	0.233
Absent (42)	3.33±0.97				4.5±1.45				72.11±7.42			

* Differentially expressed between groups, p<0.05.

RQ: relative quantification; N: number of samples; SD: standard deviation; η^2^: effect size base on Eta Squared; OP: observed power.

Dissected tumor and control specimens were quickly frozen in liquid nitrogen until nucleic acid purification. Another part of the same tissues was formalin-fixed and paraffin embedded. For the fluorescent *in situ* hybridization (FISH) assay, the remaining tumor sample was disaggregated as previously described [19].

### MYC immunoreactivity

Immunohistochemical analyses for MYC protein were performed on 125 formalin-fixed, paraffin-embedded tumor sections. Immunohistochemical staining was performed according to Calcagno *et al.* [10]. Tumor tissue sections (3 or 4 mm-thick) were deparaffinized in xylene and rehydrated in a graded series of ethanol. After heat-induced epitope retrieval, the tissue sections were incubated with primary mouse monoclonal antibody against MYC (dilution 1∶50; sc-40, Santa Cruz Biotechnology, USA and Zymed®, USA). A universal peroxidase-conjugated secondary antibody kit (LSAB System, DakoCytomation, USA) was used for the detection system. We used 3,30-diamino-benzidine/H_2_O_2_ (Dakocytomation, Denmark) as the chromogen and hematoxylin as the counterstain. Any nuclear stain with or without cytoplasmic staining was considered to be a positive result, irrespective of intensity. A MYC-positive case was defined as one having 10% or more tumor cells positive for this protein.

### Nucleic acid extraction

The genomic DNA (gDNA) was extracted using the QIAamp DNA Mini Kit (Qiagen, Germany) following the manufacturer's instructions. Total RNA was extracted with Tri-reagent® (Life Technologies, USA) according to the manufacturer's protocol. DNA and RNA concentration and quality were determined using the NanoDrop spectrophotometer (Kisker, Germany). RNA integrity was determined by gel electrophoresis (1% agarose gels). All samples were stored at −80°C until use.

### *MYC* mRNA expression

To quantitate mRNA levels of *MYC*, total RNA was isolated from 49 paired normal and tumor tissues using Trizol (Life Technologies, USA). The RNA was reverse transcribed using the High-Capacity cDNA Archive kit according to the manufacturer's protocol (Life Technologies, USA). Complementary DNA was then amplified by real-time PCR using the TaqMan probes purchased as Assays-on-demand Products for Gene Expression (Life Technologies, USA) on a 7500 Fast Real Time PCR (Life Technologies, USA). *GAPDH* gene was selected as an internal control for RNA input and reverse transcription efficiency. All real-time reverse transcription quantitative PCR (RT-qPCR) were performed in triplicate for both target gene (*MYC*: Hs00153408_m1) and internal control (*GAPDH*: NM_002046.3).

Relative quantification (RQ) of the gene expression was calculated according to Livak and Schmittgen [20]. The corresponding control sample was designated as a calibrator from each tumor.

### *MYC* copy number

FISH and qPCR were used to evaluate *MYC* copy number in a subset of 49 tumors, the same used in the study of the expression. FISH was performed according to the protocol of Pinkel *et al.* [21] with modifications introduced by Calcagno *et al.* [22]. Cells were hybridized with *Spectrum Orange* Probe (LSI Vysis/Abbott, Inc., IL) for the *MYC* gene region (8q24.12–q24.13) and nuclei were counterstained with 4′,6-diamidino-2-phenylindole antifade. Fluorescence was detected using an Olympus BX41 fluorescence microscope (Olympus, Japan) with excitation filters for 4′,6-diamidino-2-phenylindole (260 nm) and rhodamine (570 mn). For each case, 200 interphase nuclei were analyzed using an ASI image analysis system (Applied Spectral Imaging, Israel). Positive *MYC* gene signals appeared as red spots in nuclei and were scored using the criteria of Hopman *et al.* [23]. To avoid misinterpretation due to technical error, normal lymphocyte nuclei and normal gastric tissue were used as a control. The FISH results were presented as the percentage of *MYC* amplification by a cell, in which we calculated the percentage of cells showing 3 or more signals for the *MYC* probe by cell.

qPCR was performed using quantitative TaqMan CNV assays (Life Technologies, USA) for the *MYC* gene (Hs01764918_cn) and for the internal control *RNAse P* (#4403326). Multiplex qPCR reactions were performed in quadruplicate with gDNA according to the manufacturer's protocol and cycling conditions in 7500 Fast Real-Time PCR (Life Technologies, USA). The relative copy number was estimated for each sample using the Copy Caller Software V1.0 (Life Technologies, USA). Commercial human gDNAs (G1521 and G1471; Promega, USA) were used for calibration.

### *MYC* methylation

The methylation pattern and frequency of the *MYC* promoter were evaluated in 125 tumors and 67 matched control samples by Methyl-specific PCR (MSP) as previously described [24]. gDNA (2 µg) of all samples was modified by bisulfite treatment, converting unmethylated cytosines to uracils and leaving methylated cytosins unchanged [25]. Specific primers for the *MYC* promoter were as follows: F5′-TAGAATTGGATTGGGGTAAA-3′ and R5′-CCAACCAAAAATCAACATGAAT-3′ for the unmethylated reactions (expected product size of 291 bp); F5′-TAGAATTGGATCGGGGTAAA-3′ and R5′-CGACCGAAAATCAACGCGAAT-3′ for the methylated reactions (expected product size of 290 bp), as previous described [26].

PCR reactions were carried out with 0.1 µmol/L of dNTPs, 2 µmol/L of MgCl_2_, 0.5 µmol of primers, 1.25 U of Taq DNA polymerase, and 100 ng of bisulfite-modified DNA. After initial denaturation for 5 min at 94°C, 40 cycles at 9 4°C for 45 s, 52.4°C for 45 s, and 72°C for 30 s were carried out, followed by a final extension for 5 min at 72°C. PCR products were directly loaded onto 3% agarose gels and electrophoresed. The gel was stained with SYBR® Safe DNA Gel Stain (Life Technolgies, USA) and directly visualized under UV illumination. As a positive control of all MSP reactions, a gDNA sample was completely methylated using CpG Methylase (SssI, New England Biolabs, USA) following the manufacturer's instructions. Furthermore, the primers for wild-type were used to monitor complete conversion of DNA obtained in the bisulfite reaction.

Samples were stratified as: 1) hypomethylated samples when positive amplification product was detected only in the PCR with specific primers for unmethylated sequences; 2) hypermethylated samples when positive amplification was detected only in the PCR with specific primers for methylated sequences; 3) partial methylated samples when positive amplification was detected in the PCR with the two primer sets.

### *MYC* Genotyping

The three exons of the *MYC* gene were selected for mutation analysis in all 125 gastric cancer samples. The following primers were designed for PCR amplification and sequencing: exon 1 F5′-TTTATAATGCGAGGGTCTGGA-3′ and R5′-GCATTCGACTCATCTCAGCA-3′ (expected product size of 654 bp); exon 2 F5′-CTGCCTCCCGCTTTGTGT-3′ and R5′-TTTGATGAAGGTCTCGTCGT-3′ (expected product size of 423 bp), F5′-TGGGAGGAGACATGGTGAA-3′ and R5′-TGCCAATGAAAATGGGAAAG-3′ (expected product size of 507 bp); exon 3 F5′-TGTCCAGAGACCTTTCTAACGTAT-3′ and 5′-CCGTAGCTGTTCAAGTTTGTG-3′ (expected product size of 663 bp), 5′-TGTCCGTCCAAGCAGAGG-3′ and 5′-TGATGAAAACAAACAGGGATG-3′ (expected product size of 639 bp).

The PCR reactions were carried out with 0.1 µmol/L of dNTPs, 2 µmol/L of MgCl_2_, 0.5 µmol/L of primers, 1 U of Taq polymerase, and 100 ng of DNA. The PCR conditions were 95°C for 10 min, followed by 35 cycles of 1 min of denaturation at 95°C, 1 min of annealing temperature (ranging from 59 to 61°C), and 1 min of extension at 72°C. The amplicons were separated on a 2% agarose gel stained with SYBR® Safe DNA Gel Stain (Life Technolgies, USA) and directly visualized under UV illumination.

Amplicons were sequenced using the Sanger method [27]. Direct sequencing was carried out using the Big Dye® Terminatorv3.1 Cycle Sequencing kit (Life Technologies, USA) and analyzed on an ABI PRISM® 3130 Genetic Analyzer (Life Technologies, USA) using Pop 7 polymer. The in silico mutation search was performed using the Chromas Pro 1.5 (Technelysium Pty Ltd, Australia). The reference sequence was Gene ID: 4609 (NCBI). Variants with less than 1% minor allele frequency were reported. Pathogenicity of missense mutations was assessed by in silico analysis using PolyPhen (http://genetics.bwh.harvard.edu/pph/) and SIFT (http://sift.jcvi.org).

### Statistical analysis

*MYC* methylation, mutation, or its products' immunoreactivity odds ratio (OR) for clinicophatological features was estimated by logistic regression. The age at gastric tissue sampling was defined as covariate in the regression model.

The normality of distribution for quantitative variables was tested by the Shapiro-Wilk's test. Data that were not normally distributed were transformed (z-score transformation) into a normal distribution for analysis. Analysis of *MYC* mRNA expression and copy number were performed by the General Linear Model (GLM) with adjustment for age, which provides the effect size and observed power (OP) of each analysis. The effect size for GLM analyses was based on Eta Squared (η^2^), in which 0.15 and below was determined as a small effect size, 0.16–0.40 as a medium effect size, and above 0.40 as a large effect size.

The correlation between mRNA expression and copy number was analyzed by the Pearson test, in which a value of its correlation coefficient (r) below 0.30 was determined as a weak correlation, 0.30–0.70 as a medium correlation, and above 0.70 as a strong correlation.

In all analyses, the confidence interval was 95% and *p* values less than 0.05 were considered significant.

## Results

*MYC* amplification is known to be related to high protein/mRNA expression in gastric carcinogenesis [28], however, to the best of our knowledge, few studies have been performed to describe the role of methylation and *MYC* mutations in this process. To reach this goal, we analyzed 125 cases in which 68% were males and 32% were females. The mean age of our sample set was 62 years (range of 26–89 years). A slightly higher frequency of intestinal-type (56.8%) and non-cardic (58.4%) tumors were observed (Table 1 and 2).

**Figure 1:**
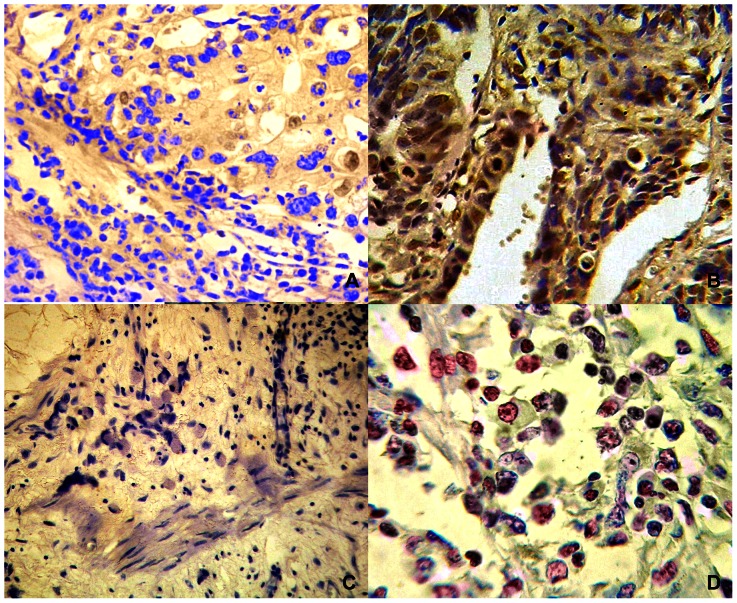
MYC protein expression. A) intestinal-type gastric cancer without MYC immunoreactivity (400×); B) intestinal-type gastric cancer presenting MYC immunoreactivity (400×); C) diffuse-type gastric cancer without MYC immunoreactivity (400×); D) diffuse-type gastric cancer presenting MYC immunoreactivity (400×).

MYC nuclear protein was found positive in 76.8% (96/125) of gastric tumors (Figure 1). MYC protein expression was more frequently observed in intestinal-type than diffuse-type tumors (*p*<0.001, OR = 7.856, CI 95% = 2.803–22.013) (Table 1). MYC immunostaining was also associated with late-onset (p = 0.026, OR = 3.276; CI 95% = 1.152–9.315), deeper tumor extension (*p* = 0.045, OR = 2.975, CI 95% = 1.027–8.623), and the presence of distant metastasis (*p*<0.001, OR = 17.682, CI 95% = 3.914–79.882) (Table 1). Futhermore, MYC immunoreactivity was associated with increased mRNA expression (*p* = 0.003, η^2^ = 0.178, OP = 0.870) and *MYC* copy number by FISH (*p* = 0.009, η^2^ = 0.139, OP = 0.759) and by qPCR (*p* = 0.003, η^2^ = 0.177, OP = 0.869) (Table 1).

The expression level of *MYC* mRNA was higher in all tumor samples than their paired controls (RQ = 3.39±0.14; range of 1.57–5.18). An increased *MYC* mRNA expression was associated with deeper tumor extension (*p* = 0.006, η^2^ = 0.152, OP = 0.801), presence of lymph node metastasis (*p* = 0.023, η^2^ = 0.107, OP = 0.632), and distant metastasis (*p*<0.001, η^2^ = 0.788, OP = 1) (Table 2). Additionally, the mRNA level was directly correlated to the *MYC* copy number (*p*<0.01; r = 0.716).

**Figure 2:**
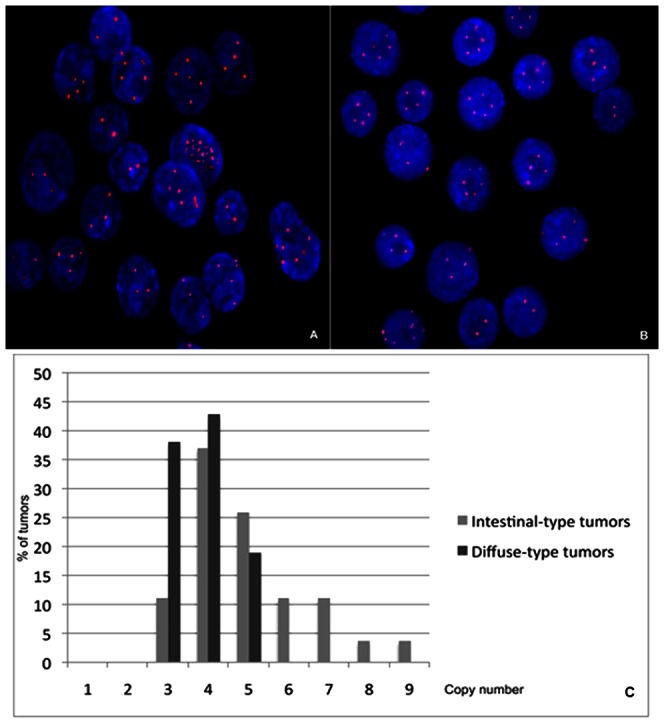
*MYC* amplification in gastric cancer. A) interphase nuclei presenting *MYC* amplification (red) in intestinal-type gastric cancer; B) interphase nuclei presenting *MYC* amplification (red) in diffuse-type gastric cancer; C) *MYC* copy number distribution by qPCR in intestinal-type and diffuse-type tumors.

Gain of *MYC* copies was found in all gastric adenocarcinoma samples by FISH and qPCR assays. By FISH, the mean percentage of cells presenting *MYC* amplification was 72.1% (range of 50 to 83.5% cells with amplification) (Figure 2 A and B). The mean of *MYC* copies by qPCR was 4.5 (range 3 to 9 copies) (Figure 2 C).

FISH and qPCR analyses showed that increased *MYC* copy number was associated with late-onset (*p*<0.001; η^2^ = 0.257, OP = 0.976; *p* = 0.025; η^2^ = 0.103, OP = 0.662, respectively ), intestinal-type cancer (*p* = 0.037; η^2^ = 0.091, OP = 0.557; *p* = 0.009; η^2^ = 0.139, OP = 0.762, respectively), and the presence of distant metastasis (*p* = 0.001; η^2^ = 0.221, OP = 0.942; *p*<0.001; η^2^ = 0.356, OP = 0.999, respectively). In addition, *MYC* amplification by FISH was associated with advanced tumor stages (*p* = 0.037; η^2^ = 0.091, OP = 0.558), however, only two early tumors were analyzed in this subset of samples (Table 2).

**Figure 3:**
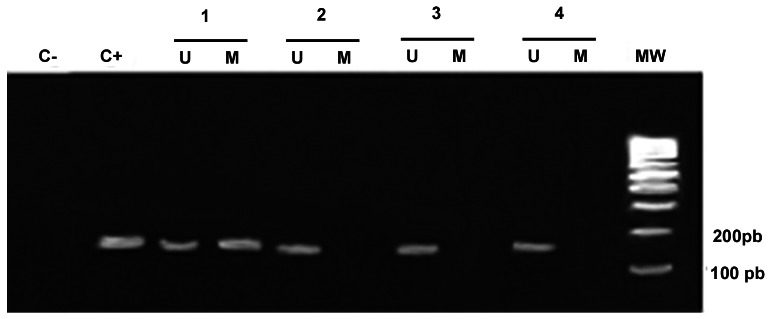
Methylation analysis of the *MYC* promoter showing methylated and unmethylated bands. Sample 1 presented partial methylation. Samples 2, 3 and 4 presented a hypomethylated promoter. C-: blank; C+: positive control, gDNA sample completely methylated; U: unmethylated; M: methylated: MW: molecular weight marker; bp: base pairs.

All gastric cancer samples presented positive amplification with an unmethylated primer set. Interestingly, 86.4% of cancer samples were hypomethylated. On the other hand, the presence of unmethylated sequences at the *MYC* promoter was observed in 28.4% of control samples (partial methylated samples), suggesting the loss of methylation in these samples (Figure 3). The primers's specificity and MSP results were confirmed using the bisulfite sequencing PCR (BSP) approach [29] in which we randomly selected five hypomethylated samples; five hypermethylated samples and five partial methylated samples (data not shown). *MYC* hypomethylation was more frequently observed in the diffuse-type as compared to the intestinal-type gastric cancer (*p* = 0.007; OR = 8.554; 95% CI = 01.798–40.695, using diffuse-type as reference group). In addition, *MYC* hypomethylation was associated with advanced tumor stages (*p* = 0.033; OR = 6.602; 95% CI = 1.162–37.501), deeper tumor extension (*p* = 0.022; OR = 4.752; 95% CI = 1.257–17.965), and the presence of lymph node metastasis (*p* = 0.032; OR = 5.12; 95% CI = 1.149–22.814) (Table 1).

With regards to gene sequencing, no novel mutation was detected in gastric tumors. Thirteen (10.4%) tumor samples presented at least one known mutation, with variants on less than 1% minor allele frequency. In total, 18 mutations were identified, with 4 samples exhibiting co-occurring mutations. In exon 1, five with GG and four with CG at rs117856857; one with GG at rs73707292; and four with CT at rs4645949. In exon 2, concerning missense mutations, two tumors harbored a mutation at codon 47 resulting in a change from tyrosine to histidine (rs114570780; SIFT prediction = deleterious; PolyPhen prediction = probably damaging), and two at codon 72 resulting in a change from proline to serine (rs28933407; SIFT prediction = tolerated; PolyPhen prediction = probably damaging). All tumor with a mutation in exon 2 presented one or two known mutation in exon 1. Exon 2 mutations were only detected in diffuse-type tumors in advanced stage. No mutation was identified in exon 3. The presence of known *MYC* mutations was associated with diffuse-type tumors (*p* = 0.004, OR = 21.717, 95% CI = 2.678–176.111; using diffuse-type as reference group) and the presence of distant metastasis (*p* = 0.032, OR = 4.492, 95% CI = 1.141–17.679).

## Discussion

The MYC protein has an effect on about 15% of the genes in the human genome [30]. Thus, MYC deregulation may result in alterations in different biological pathways involved in cancer initiation and progression [5]. However, up to date, the relationship between *MYC* alterations and clinicopathological parameters has not been well understood. Our samples presented a male-female ratio of 2∶1 and the majority of the patients were older than fifty-five. Moreover, the intestinal-type gastric cancer was more frequent than the diffuse-type and tumors were more frequent in non-cardia. These epidemiological data are in accordance with previous studies [28], [31]–[33].

Chromosomal translocations in the *MYC* locus is very commun in haematopoietic cancers. However, in solid human tumours like gastric cancer, *MYC* alterations are commonly due to gene amplification [34]. Furthermore, *MYC* is recognized to be the most frequently amplified protein-coding gene across all cancer types [35]. In the present study, we observed three or more *MYC* gene copies in all gastric tumors studied, corroborating with previous studies in primary gastric tumors of individuals from our population [10], [28], [36]–[39], as well as from Eastern Asia and Europe [40]–[42]. Also, *MYC* amplification was observed in plasma [43] and in gastric cancer cell lines established in our group [44]–[47]. Additionally, our group had observed that clonal high amplification of *MYC* is less frequent in diffuse-type than in intestinal-type primary gastric cancer [10], [22], [28], and this was reinforced by this study.

MYC overexpression is described as ranging from 15.6% to 100% in primary gastric cancers [9]. *In vitro* studies with knocked-down MYC expression in gastric cancer cell lines demonstrated the crucial role of MYC expression in gastric tumor cell growth, survival, and the maintenance of tumor cell parameters that may contribute to malignant potential [48]. Moreover, Mehndiratta *et al.*, [49] showed a significant decrease (40%) in MYC expression of both mRNA and protein and its downstream targets using siRNA.

In the present study, 76.8% of gastric tumors showed MYC immunoreactivity and all tumors, intestinal and diffuse type, presented increased mRNA expression compared to their paired controls. Furthermore, we observed that MYC immunoreactivity and increased mRNA expression were associated with deeper tumor extension and presence of metastasis. These findings suggest that MYC has a role in the tumor invasiveness, metastasis, and thus aggressiveness, corroborating a previous study [37]. Although some analyses presented a small effect size, these findings are also in agreement with a previous study of our group in non-human primates, in which we demonstrated a continuous increase of *MYC* mRNA expression and copy number during the sequential steps of intestinal-type gastric carcinogenesis in N-methyl-nitrosourea (MNU)-treated non-human primates [16]. On the other hand, any association between MYC and histological grade, tumor location, lymph node metastasis, or pathological stage was detected in a gastric cancer study developed in a Chinese population [50], reinforcing that the ethnicity of the afflicted population may lead to biologically and clinically gastric cancer subsets [51].

Although, gene amplification is not necessarily associated or required for its overexpression, our study shows that MYC immunoreactivity, *MYC* mRNA levels, and copy number were directly correlated.

DNA methylation is a potent mechanism of transcriptional repression. Proper genomic methylation-patterns become profoundly altered in cancer cells: both gains (hypermethylation) and losses (hypomethylation) of methylated sites have been observed [52], [53]. Hypomethylation at specific promoters can activate the aberrant expression of oncogenes and loss of imprinting in some loci [44]. So far, few studies discussed the relationship between the methylation pattern of *MYC* and its effect on gene expression and on carcinogenic processes. *MYC* hypomethylation was previously associated with the oncogenic progression and metastasis induction in a rat model of liver cancer [54] and in human colorectal cancer samples [55]. In addition, *MYC* hypomethylation was also associated with *MYC* expression in gastric tumors [26], [56]–[58] and cell lines [48]. However, we were unable to observe a significant association between *MYC* hypomethylation and its expression in our samples. Among other factors, this lack of association may be due to the presence of *MYC* amplification in all tumors with hypomethylated promoters (86.4% of samples) and thus, masking the possible effect of this epigenetic modification on *MYC* transcriptional regulation.

Suzuki *et al.* [59] previously showed that a high level of hypomethylation was an indicator of poor prognosis in both gastric and colon cancer, and epigenetic alterations were age dependent, occuring before genetic alterations. In the present study, some controls already presented unmethylated sequences in the *MYC* promoter. In addition, we demonstrated that *MYC* hypomethylation was associated with a more aggressive phenotype (tumor aggressiveness, presence of lymphnode metastasis, and histological types of cancer). These findings suggest that *MYC* demethylation may be accumulated during tumor progression and this could be a common event in gastric carinogenesis [60], [61], since this mechanism has been observed for other genes in several tumor types [62], [63]. As already proposed for DNA hypermethylation [64], the promoter hypomethylation may be used as a new generation of biomarkers and holds diagnostic and prognostic promise for clinicians.

To the best of our knowledge, *MYC* gene exons have never been completely sequenced in human gastric tumors. Here, we sequenced the three exons of *MYC*: exon 1 is a non-coding protein, exons 2 and 3 are protein-coding [for review see (Pelengaris & Khan, 2003)]. We did not find any new mutation, however, we observed that 4 tumors presented missense mutations (rs114570780 and rs28933407) on exon 2. These mutations were in an evolutionary conserved sequence of *MYC*: the transactivation domain [4], [65]. In addition, both identified mutations were considered as probably damaging according to the PolyPhen software. The change of proline to serine at codon 72 was also previously reported as a pathogenic variant in the NCBI dbSNP database (http://omim.org/). Thus, both mutations at exon 2 may affect MYC activity in gastric tumors. In addition, the presence of *MYC* mutations was associated with distant metastasis. However, further investigations are necessary to clarify if *MYC* mutations have a role in the metastatic process.

According to Laurén classification, gastric adenocarcinoma is classified mainly into intestinal and diffuse types [17]. Intestinal-type gastric cancer progresses through a number of sequential steps, beginning with atrophic gastritis followed by intestinal metaplasia, intraepitelial neoplasia, and carcinoma [66]. On the other hand, the diffuse-type generally does not evolve from precancerous lesions [67], [68]. In the present study, we observed that the intestinal-type presented more frequent MYC immunoreactivity, as well as a higher number of *MYC* copies than diffuse-type tumors, which corroborates with previous studies of our group [10], [22], [28], [38]. In addition, *MYC* hypomethylation and point mutations were more frequently observed in diffuse-type as compared to intestinal-type tumors. Thus, our findings support that these two histological subtypes follow different genetic pathways and may be two distinct entities [67].

In conclusion, our data suggest that *MYC* overexpression and promoter hypomethylation may have a role in the gastric carcinogenesis process. *MYC* deregulation was associated mainly to poor prognostic features. Our results also reinforce the presence of different pathways involved in intestinal-type and diffuse-type gastric carcinogenesis. Thus, our findings suggest that MYC may be a useful marker for clinical stratification and prognosis.
